# *Mycobacterium fortuitum* osteomyelitis of the cuboid bone treated with CERAMENT G and V: a case report

**DOI:** 10.5194/jbji-7-163-2022

**Published:** 2022-07-25

**Authors:** Kilian Fraga, Miriam Maireles, Marc Jordan, Laura Soldevila, Oscar Murillo

**Affiliations:** 1 Department of Orthopedic Surgery and Traumatology, Bellvitge University Hospital,08907 Hospitalet de Llobregat, Barcelona, Spain; 2 Septic Unit, Bellvitge University Hospital, 08907 Hospitalet de Llobregat, Barcelona, Spain; 3 Department of Infectious Diseases, Bellvitge University Hospital, 08907 Hospitalet de Llobregat, Barcelona, Spain

## Abstract

We present the rare case of a 61-year-old female with *Mycobacterium fortuitum* osteomyelitis of the cuboid bone following penetrating plantar trauma. The patient underwent a single-stage surgery for the condition, including lesion debridement and bone defect filling with absorbable, gentamicin-/vancomycin-loaded, calcium sulfate–hydroxyapatite biocomposites, that resolved favorably 5 months after intervention.

## 1 Introduction

*Mycobacterium fortuitum* (*M. fortuitum*) is a fast-growing, gram-positive, opportunistic human pathogen belonging to the nontuberculous mycobacteria (NTM) family that is commonly found in water and soil. NTM infection of the musculoskeletal tissue is a rare disease that may occur in both immunocompetent and immunocompromised individuals; moreover, it is commonly missed or its diagnosis is delayed due to the absence of systemic symptoms and the difficulty involved with isolating the pathogen (Wong et al., 2020; Kasperbauer and Daley, 2020).

The management of NTM osteomyelitis usually includes a combination of systemic and local antimycobacterial therapy and surgical debridement. Several surgical procedures are available to deliver antibiotics locally and manage bone defects, with these treatments mainly differing with respect to the material used to fill the defect, such as autologous bone grafting or antibiotic-loaded cement-based grafts (Wassif et al., 2021). Although a few reports have described *M. fortuitum* osteomyelitis of the cuboid bone (Vasiliadis et al., 2021; Miron et al., 2000; Wong et al., 2020), this is the first case, to our knowledge, in which bone defect restoration has been performed with a single-stage treatment using absorbable, gentamicin- and vancomycin-loaded, calcium sulfate–hydroxyapatite biocomposites.

**Figure 1:**
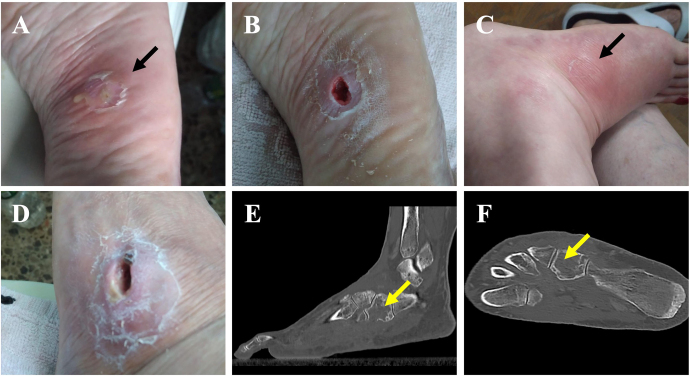
A plantar foot abscess following a puncture injury before **(a)** and after **(b)** debridement. The subsequent appearance of a dorsolateral fistula after failed antibiotic treatment before **(c)** and after **(d)** debridement. **(e, f)** A CT scan showing cuboidal osteomyelitis with medullary bone destruction (yellow arrows).

**Figure 2:**
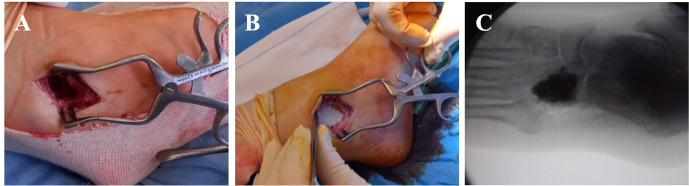
**(a)** Intraoperative cuboid bone debridement. **(b)** The medullary defect was filled with absorbable, gentamicin- and vancomycin-loaded, calcium sulfate–hydroxyapatite biocomposites (CERAMENT G and V). **(c)** An anteroposterior X-ray after surgery.

## 2 Case presentation

An immunocompetent 61-year-old female presented to the emergency department of our hospital with a cutaneous abscess on the left foot. She was well until 3 months before admission, when she accidentally stepped on a nail in her yard causing a puncture wound to the plantar surface of her foot. The patient was initially managed at a primary health center, where she presented with swelling, tenderness, and redness of the skin on the plantar aspect of the mid-foot. She was diagnosed with cellulitis, and oral antibiotic therapy with levofloxacin (500 mg d ${}^{-1}$ ) and clindamycin (450 mg every 8 h) was initiated for 8 weeks. Initial improvement was noted, but the condition relapsed 3–4 weeks after the completion of treatment, and a plantar abscess appeared (Fig. 1a). On admission to our hospital, the abscess was debrided (Fig. 1b), and samples were collected for microbiological analysis. Laboratory blood tests revealed elevated levels of C-reactive protein (CRP; 99.1 mg L ${}^{-1}$ ), and bacterial cultures returned the presence of *Mycobacterium fortuitum*. After antimicrobial susceptibility results became available (Table 1), the antibiotic regimen was changed to oral levofloxacin (750 mg d ${}^{-1}$ ) and clarithromycin (500 mg every 12 h). Although infection of the skin and the underlying plantar soft tissue evolved favorably, the patient reported increased pain and inflammatory signs, and a fistula appeared in the dorsum of her foot 1.5 months after antibiotic therapy was initiated (Fig. 1c). The fistula was debrided (Fig. 1d), and samples were collected for bacterial culture. The results again showed the presence of *M. fortuitum* (Table 1); thus, a standing computed tomography (CT) scan was requested. CT findings were consistent with cuboid osteomyelitis with medullary destruction (Fig. 1e, f). Surgical intervention was then decided upon, and surgery was performed 1 month later (i.e., 2.5 months after the initiation of the antibiotic treatment in our center and 5.5 months after plantar puncture). Surgery consisted of the radical debridement of the medullary portion of the cuboid bone to remove all infected and necrotic tissue until healthy bone was exposed (Fig. 2a) as well as copious irrigation with saline solution to clean the lesion. The dead space (a large bony defect caused by debridement) was filled with two absorbable, 40 % hydroxyapatite and 60 % calcium sulfate biocomposites loaded with either gentamicin (CERAMENT G) or vancomycin (CERAMENT V) (BONESUPPORT, Lund, Sweden) (Fig. 2b). The concentration of antibiotics in the respective biocomposites was 17.5 mg of gentamicin per milliliter of CERAMENT G paste and 66 mg of vancomycin per milliliter of CERAMENT V paste. The procedure was performed by applying an initial layer (5 mL) of CERAMENT G followed by the application of a second layer (5 mL) of CERAMENT V over the former. Postoperative magnetic resonance imaging (MRI) confirmed the correct filling of the defect with the bone graft substitute (Fig. 2c). Following surgery, the patient was started on a 3-month regimen of oral linezolid (600 mg d ${}^{-1}$ ) and doxycycline (100 mg every 12 h), in combination with IV imipenem (1 g every 12 h) for the first month only. Intraoperative cultures, including cultures with mycobacteria-specific media, did not identify *M. fortuitum*, and no other bacteria were isolated.

**Table 1:** Antimycobacterial susceptibility testing and results determined by manual microdilution from cultures obtained from the debridement of the plantar abscess and the subsequent fistula on the dorsum of the foot.

	*Mycobacterium fortuitum* susceptibility			
	Plantar abscess		Dorsolateral fistula ${}^{1}$	
Amikacin	S	≤1 µg mL ${}^{-1}$	S	≤1 µg mL ${}^{-1}$
Cefoxitin	S	≤4 µg mL ${}^{-1}$	I	32 µg mL ${}^{-1}$
Ciprofloxacin	S	1 µg mL ${}^{-1}$	I	2 µg mL ${}^{-1}$
Clarithromycin	S	1 µg mL ${}^{-1}$	S	1 µg mL ${}^{-1}$
Co-trimoxazole ${}^{2}$	S	2/38 µg mL ${}^{-1}$	R	8/152 µg mL ${}^{-1}$
Doxycycline	S	≤0.12 µg mL ${}^{-1}$	S	≤0.12 µg mL ${}^{-1}$
Imipenem	S	≤2 µg mL ${}^{-1}$	S	≤2 µg mL ${}^{-1}$
Linezolid	S	≤1 µg mL ${}^{-1}$	I	≤16 µg mL ${}^{-1}$
Moxifloxacin	S	≤0.25 µg mL ${}^{-1}$	S	≤0.25 µg mL ${}^{-1}$
Tobramycin	S	4 µg mL ${}^{-1}$	S	4 µg mL ${}^{-1}$

The abbreviations used in the table are as follows: S – susceptible, I – intermediate, and R – resistant. ${}^{1}$ Susceptibility tests from the samples of the dorsolateral fistula were performed 1.5 months after those from the plantar abscess. ${}^{2}$ Co-trimoxazole, the combination of trimethoprim (TMP) and sulfamethoxazole (SMX), is presented as TMP/SMX in the table.

**Figure 3:**
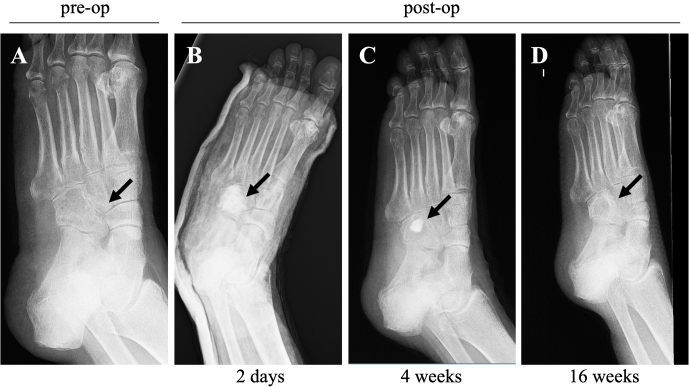
X-ray (medial oblique view) images taken before and after the surgery: **(a)** an X-ray image taken before surgery, and X-ray images taken at 2 d **(b)**, 4 weeks **(c)**, and 16 weeks **(d)** post-surgery, showing the resolution of inflammatory and infectious parameters, correct healing, and the evolution of soft tissues.

The subsequent evolution of the condition was satisfactory, with complete resolution of the inflammatory symptoms (CRP levels were down to 7.4 mg L ${}^{-1}$ ), soft tissue wound healing, and tolerance to rehabilitation. Radiographs taken 4 months after the surgery (Fig. 3) showed increased cuboid bone formation and active remodeling of the biomaterial into medullary bone. A total of 5 months after the surgery, the patient tolerated full-load ambulation without limitations, and the infectious parameters remained negative for more than 3 months after the cessation of antibiotic treatment.

## 3 Discussion

The diagnosis and treatment of chronic post-traumatic and postoperative osteomyelitis remain a challenging clinical problem. Treatment usually consists of surgical debridement and combined long-term systemic antibiotic therapy and local antibiotic delivery. Surgery is indicated in the case of antibiotic therapy failure or chronic osteomyelitis. Debridement aims (1) to remove all infected or necrotic bone and soft tissue until bleeding healthy tissue is reached and (2) to reduce the bacterial load at the site of infection, improving the effect of antibiotics (Dinh et al., 2009). This is particularly relevant for *M. fortuitum* osteomyelitis, as fast-growing mycobacteria are usually more difficult to treat than slow-growing mycobacteria because of their inherent drug resistance (Kasperbauer and Daley, 2020). In this report, the combined administration of systemic antibiotics (linezolid, doxycycline, and imipenem) and locally released gentamicin and vancomycin from CERAMENT carriers proved to be clinically effective to treat *M. fortuitum* cuboid bone osteomyelitis. The systemic antibiotic selection was based on the isolated pathogen and the results of susceptibility testing, avoiding monotherapy to prevent antibiotic resistance. Regarding local antibiotics, the inhibitory activity of gentamicin against *M. fortuitum* has been previously reported, with MIC90 values ranging from 16 to 32 µ g mL ${}^{-1}$ (Kamada et al., 2021; Shen et al., 2018). Due to the fact that (1) the gentamicin concentration in CERAMENT G (17.5 mg mL ${}^{-1}$ ) is roughly 1000 times higher than the MIC90 and (2) the zero-order release kinetics of CERAMENT, a clinically effective antibiotic concentration was expected for a long period of time. As for vancomycin, although *M. fortuitum* is resistant to this antibiotic, we considered that the combined use of both gentamicin and vancomycin-loaded biocomposites could be a good strategy to deal with *M. fortuitum* while also preventing the local appearance of bacterial superinfection such as methicillin-resistant *Staphylococcus aureus* (MRSA).

Debridement of the infected bone may leave a large bony defect (dead space) that must be repaired, as any bone void remaining after debridement will provide a poorly vascularized environment that leaves the patient predisposed to the recurrence of infection; moreover, this bone void is a potential weakness that increases fracture risks (Ferguson et al., 2019). Different methods exist to manage dead space, which are all aimed at replacing necrotic bone and scar tissue with durable vascularized tissue (Wassif et al., 2021). Antibiotic-impregnated poly(methyl methacrylate), PMMA, offers high local antibiotic concentrations and provides immediate structural stabilization (Luo et al., 2016). However, PMMA beads exhibit a burst antibiotic release and, consequently, subtherapeutic release kinetics, with the levels of eluted antibiotic declining over time. Moreover, PMMA carriers are nonbiodegradable; thus, they require secondary surgery for removal, as they prevent bone ingrowth, thereby increasing hospital stay and costs. In contrast, biodegradable and bioabsorbable antibiotic-impregnated materials enable single-stage surgery and provide zero-order release kinetics, thereby preventing the growth of antibiotic-resistant bacterial strains due to a lack of long-term and subtherapeutic concentration tail release (Luo et al., 2016). CERAMENT is a bioabsorbable ceramic cement consisting of 60 % calcium sulfate (CS) and 40 % hydroxyapatite (HA). Fast-resorbing CS serves as a carrier for the antibiotic and the highly osteoconductive HA. CS undergoes early dissolution, ensuring an early and constant release of high antibiotic levels while leaving behind a more slowly dissolving HA porous scaffold, which provides more prolonged structural stability and bone ingrowth (Ferguson et al., 2019). The optimized HA / CS ratio is designed to allow CERAMENT to resorb at the same rate that bone forms (Horstmann et al., 2018). The effectiveness of CERAMENT G and V in the treatment of osteomyelitis has been demonstrated in several reports (McNally et al., 2016; Hofmann et al., 2020; Niemann et al., 2022); however, authors have also reported drawbacks. For instance, McNally et al. (2016) reported prolonged wound leakage in 6 % of their cohort for up to 11 weeks after surgery, and Niemann et al. (2022) described wound dehiscence and prolonged secretion in 30 % of their patients. In this case report, radiographic images taken at the 4-month follow-up show that the bone void filler material was reabsorbed and new bone was formed, indicating that CERAMENT G and V are an appropriate therapeutic option for *M. fortuitum* osteomyelitis.

## 4 Conclusions

Osteomyelitis due to *Mycobacterium fortuitum* is a rare condition that requires combined surgical and antibiotic treatment. Our results show that the application of absorbable, gentamicin- and vancomycin-loaded, calcium sulfate–hydroxyapatite biocomposites to the bone defect combined with systemic antibiotic treatment causes the remission of *M. fortuitum* infection and subsequent new bone formation while avoiding the need for another graft supply intervention. Further studies will be required to assess the superiority of CERAMENT G and V biocomposites over other current methods for the treatment of NTM osteomyelitis.

## Data Availability

No data sets were used in this article.
